# *QuickStats:* Rates* of Firearm-Related Deaths^†^ Among Persons Aged ≥15 Years, by Selected Intent^§^ and Age Group — National Vital Statistics System, United States, 2019

**DOI:** 10.15585/mmwr.mm7010a5

**Published:** 2021-03-12

**Figure:**
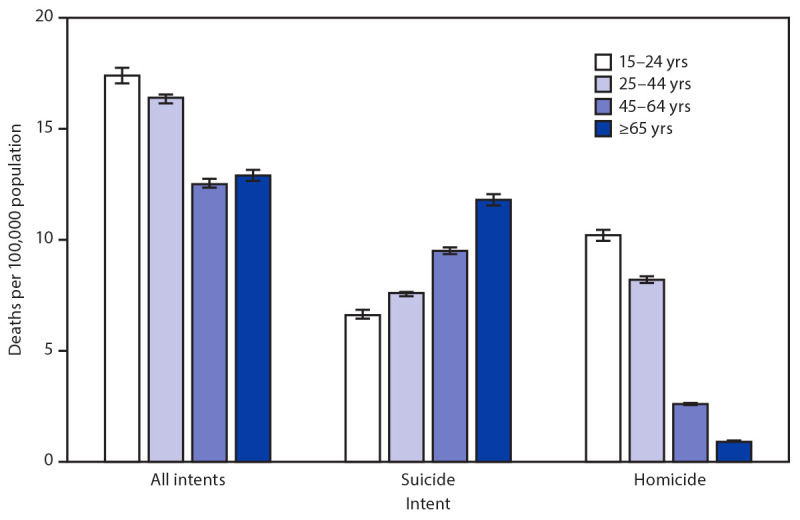
Among persons aged ≥15 years, for all firearm-related deaths (all intents), rates were highest among those aged 15–24 years (17.4 per 100,000). For deaths involving firearm-related suicides, rates increased with age, from 6.6 among persons aged 15–24 years to 11.8 among those aged ≥65 years. A different pattern was found for firearm-related homicides, in which rates decreased with age, from 10.2 among those aged 15–24 years to 0.9 among those aged ≥65 years.

**Source:** National Center for Health Statistics, National Vital Statistics System, Mortality Data, 2019. https://www.cdc.gov/nchs/nvss/deaths.htm

